# Tobler’s law and wavefront patterns in the spatial spread of COVID-19 across Europe during the Delta and Omicron waves

**DOI:** 10.1177/14034948221141806

**Published:** 2022-12-15

**Authors:** Thomas Plümper, Eric Neumayer

**Affiliations:** 1 Department of Socioeconomics, Vienna University of Economics and Business, Austria; 2 Department of Geography and Environment, London School of Economics and Political Science (LSE), UK

**Keywords:** Infectious disease, SARS-CoV-2, COVID-19, geography, Tobler’s law, wavefront model

## Abstract

**Aims:**

Epidemic wavefront models predict the spread of medieval pandemics such as the plague well. Our aim was to explore whether they contribute to understanding the spread of COVID-19, the first truly global pandemic of the 21st century with its fast and frequent international travel links.

**Methods:**

We analysed the spatial spread of reaching a threshold of very high incidence of new daily infections of the virus across European countries in the autumn of 2021 in which the Delta variant was dominant, as well as an even higher threshold of incidence in the subsequent spread of infections across the same set of countries during the winter of 2021/2022 when the Omicron variant of the virus became dominant.

**Results:**

We found patterns that are consistent with wavefront models for both periods of the pandemic in Europe.

**Conclusions:**

**Modern means of transportation strongly accelerated the spread of the virus and typically generated diffusion patterns along bidirectional constrained mobility networks in addition to stochastic diffusion processes. However, since the majority of mobility, including mobility across international borders, is over short distances, wavefront patterns in the spread of a pandemic are still to be expected.**

## Introduction

Tobler’s well-known first law of geography claims that ‘everything is related to everything else, but near things are more related than distant things’ [1]. Local networks are denser and lead to more social contact between infected and susceptible people than networks that span longer distances. This logic gives rise to an old epidemiological diffusion model called the wavefront model [2]. In its deterministic variant [3,4], the wavefront model assumes a single access point of a virus into a community from where it spreads in all directions at constant velocity [5]. Deterministic wavefront models neglect stochastic spread as well as spread along ‘bidirectional constrained mobility networks’ [6] – for example, along popular airline routes, train networks and highways, which cross international borders and render it possible that infections transmit over long distances. These different spatial dynamics are of course not mutually exclusive.

Wavefront models predict the diffusion patterns of the plague, cholera and Ebola in Sierra Leone [7,8] well, but with modern fast transportation modes, these simple wavefront models no longer appear to explain the spread of infectious diseases in modern societies appropriately [6,9]. We provide evidence that the diffusion pattern of SARS-CoV-2 across European countries during the Delta and the Omicron waves resembles the predictions of stochastic wavefront models, albeit not with constant velocity.

Our analysis differs from classical models explaining the spatial spread of infectious diseases, since we do not consider the wavefront of the virus itself [10]. After the first wave of the pandemic, there have been local infections with no frontline that separates regions the virus has reached from regions the virus has not yet reached. Instead, we analyse when an ensuing wave of infections surpasses a defined threshold population-standardized incidence rate at the country level.

## Methods

Few European countries have the capacity and willingness for extensive genome sequencing, with all but six of them sequencing <2.5% of tests during December 2021 [11] In the UK – a country with one of the more comprehensive and thus reliable sequencing strategies – the Omicron variant replaced the Delta variant in about four weeks [12]. Since the replacement process did not start simultaneously in all countries of our sample and since many countries have not sequenced enough tests, we are not able to distinguish precisely between an increase in incidence caused by Delta and an increase caused by Omicron for December 2021. Still, most European countries experienced two distinguishable waves in late autumn 2021 and early winter 2021/2022, and for simplicity reasons, we label them the Delta and Omicron waves, although of course, some of the infections have been caused by other variants.

To account for the higher infection rate of Omicron relative to Delta [13], we employed two different thresholds of incidence of infections, with data sourced from Our World in Data [14]. Specifically, we used thresholds of 300 and 1000 new daily infections per million people for the Delta and Omicron waves, respectively, smoothed over a period of one week, showing the weeks in which countries reached these thresholds on a map. Admittedly, these thresholds are somewhat arbitrary. Choosing slightly higher or lower thresholds does not have a major impact on our results.

## Results

**Figure 1.**
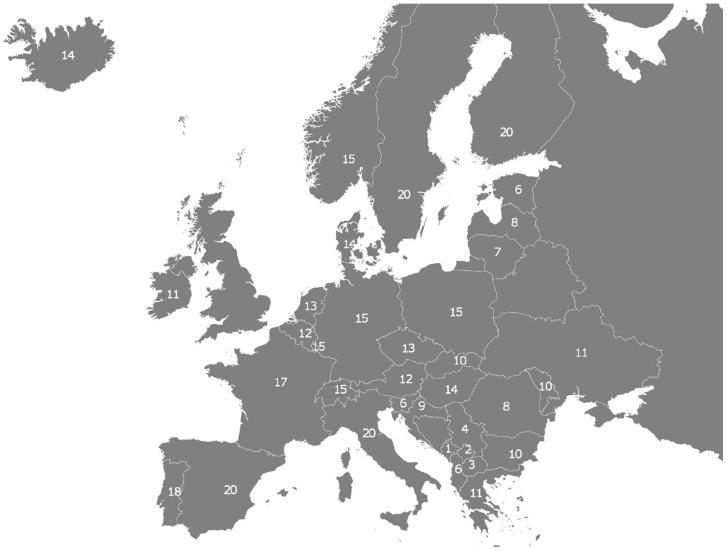
The diffusion map of the Delta wave in Europe, 2 August 2021 (week 1) to 13 December 2021 (week 20). Note: number denotes week in which country crosses threshold of 300 new daily infections per million people.

Figure 1 displays when European countries reached the threshold of 300 new daily infections per million people. Each week starts on Monday and ends on Sunday, and we denote the week of 2–8 August 2021, in which Montenegro was the first to reach the threshold, by ‘1’. Finland, Sweden, Italy and Spain were the last to reach this threshold in the week of 13–19 December 2021 or week 20.

Figure 1 provides evidence for an epidemic wavefront movement of SARS-CoV-2 infections during this period. After Montenegro’s lead in week 1, other neighbouring countries on the Balkans reached the high incidence threshold only a few weeks after. The wavefront then spreads further north-westwards to Croatia and Slovenia as well as north-eastwards to Romania, Bulgaria, Moldova and Ukraine. By week 11, all countries in Southeast Europe but also in the far eastern part of the continent, bar Finland, had crossed the threshold. The wavefront then proceeds to move in a more north-western direction, reaching Central Europe and the Benelux states as well as Iceland, Denmark and Norway by week 15. From there, it moves eastwards to the other two Scandinavian countries but also south and south-westwards to France and Southern Europe. Note that according to officially reported data, Bosnia-Herzegovina, Russia and Belarus never crossed the threshold during this period. This could be either due to poor surveillance or because of deliberate attempts to downplay the severity of the pandemic by misreporting data [15]. Relatedly, it is also possible that the Baltic countries reaching the threshold earlier than what the wavefront model predicts is due to infections spilling over from neighbouring Belarus and Russia. Alternatively, it could also be caused by other processes, including stochastic ones, which may also account for why Norway reached the threshold five weeks before Sweden and Finland did.

**Figure 2.**
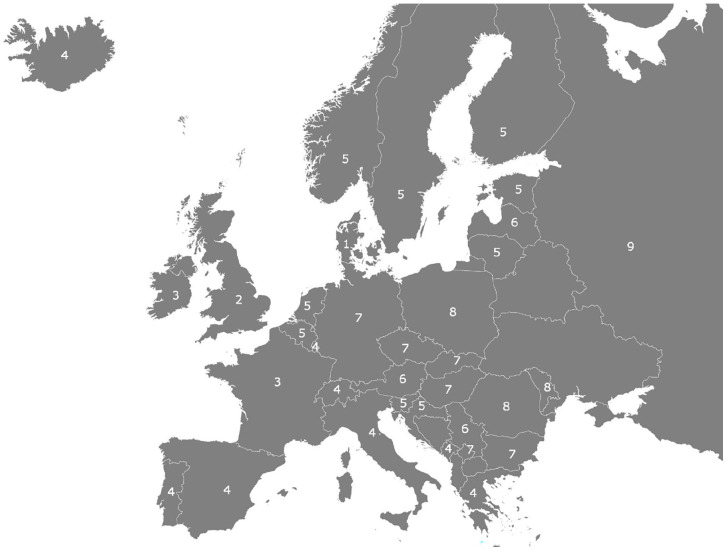
Diffusion map of the Omicron wave in Europe, 6 December 2021 (week 1) to 31 January 2022 (week 9). Note: number denotes the week in which country crosses threshold of 1000 new daily infections per million people.

Figure 2 displays when European countries reached the threshold of 1000 new daily infections per million people. As before, each week start on Monday and ends on Sunday, and we denote the week of 6–12 December 2021 in which Denmark was the first to reach the threshold, by ‘1’. Russia was the last to reach this threshold in the week commencing 31 January 2022 or week 9.

Figure 2 also provides evidence for an epidemic wavefront movement of SARS-CoV-2 infections during this period, albeit at a much faster rate. Starting in Denmark, the next countries to reach the threshold were the UK, Ireland and France. From there, it moved south and east to Luxembourg, Italy, Spain, Switzerland, Portugal and Montenegro. By week 5, the wavefront pushes the remaining Benelux countries and the remaining Nordic countries, including the Baltic countries, over the threshold, except for Latvia which was five days behind Estonia and Lithuania. In weeks 7 and 8, the wave reached Central and Eastern European countries and finally Russia in week 9. As of 28 February 2022, only five European countries had not reached the threshold of 1000 daily cases per million, smoothed over a one-week period. As mentioned above for the Delta wave, for two of them, namely Belarus and Bosnia-Herzegovina, official infection data are unreliable. Albania, North Macedonia and Ukraine peaked at just below 800, 845 and 860 daily cases per million in mid-January, the end of January and early February, respectively.

## Conclusions

An understanding of epidemic diffusion patterns can help public health officials to predict and therefore prepare for when and from where a significant increase in infections is likely to occur. To our knowledge, this is the first paper providing evidence for the existence of wavefront diffusion patterns during the Delta and the Omicron waves of the COVID-19 pandemic and that wavefront models still contribute to understanding the spread of infectious diseases such as SARS-CoV-2. Naturally, spread also occurs stochastically and along bidirectional constrained mobility network patterns, including seeded infections imported from far-away places facilitated by international air travel such that non-proximate spread occurs simultaneously to proximate wavefront-type spread [16]. There are also idiosyncrasies of national policymaking [17]. For example, the UK never fell below the threshold of 300 new infections per million people after the lifting of most public health restrictions in England on 19 July 2021.

Tobler’s law is not the only possible explanation for the observable empirical patterns in Figures 1 and 2: near people are not only more connected than geographically distant people, they also tend to be more similar. If this holds, then the similar epidemiological dynamic in geographically proximate countries could also be triggered by similar behavioural patterns and similar policies in neighbouring countries, including travel restrictions, similar vaccination efforts and the like. This should prove fruitful for future research to explore.
